# Molecular Variability and Distribution of *Sugarcane Mosaic Virus* in Shanxi, China

**DOI:** 10.1371/journal.pone.0151549

**Published:** 2016-03-17

**Authors:** Xiansheng Xie, Wei Chen, Qiang Fu, Penghui Zhang, Tianci An, Aimin Cui, Derong An

**Affiliations:** 1 State Key Laboratory of Crop Stress Biology for Arid Areas and College of Plant Protection, Northwest A&F University, Yangling, Shaanxi, China; 2 Wheat Research Institute, Shanxi Academy of Agricultural Sciences, Linfen, Shanxi, China; 3 College of Life Science, Shanxi Normal University, Linfen, Shanxi, China; Oklahoma State University, UNITED STATES

## Abstract

**Background:**

*Sugarcane mosaic virus* (SCMV) is responsible for large-scale economic losses in the global production of sugarcane, maize, sorghum, and some other graminaceous species. To understand the evolutionary mechanism of SCMV populations, this virus was studied in Shanxi, China. A total of 86 maize leaf samples (41 samples in 2012 and 45 samples in 2013) were collected from 4 regions of Shanxi.

**Results:**

Double-antibody sandwich (DAS)-ELISA and RT-PCR showed 59 samples (30 samples in 2012 and 29 samples in 2013) to be positive for SCMV, from which 10 new isolates of SCMV were isolated and sequenced. The complete genomes of these isolates are 9610 nt long, including the 5′ and 3′ non-coding regions, and encode a 3063-amino acid polyprotein. Phylogenetic analyses revealed that 24 SCMV isolates could be divided on the basis of the whole genome into 2 divergent evolutionary groups, which were associated with the host species. Among the populations, 15 potential recombination events were identified. The selection pressure on the genes of these SCMV isolates was also calculated. The results confirmed that all the genes were under negative selection.

**Conclusions:**

Negative selection and recombination appear to be important evolutionary factors shaping the genetic structure of these SCMV isolates. SCMV is distributed widely in China and exists as numerous strains with distinct genetic diversity. Our findings will provide a foundation for evaluating the epidemiological characteristics of SCMV in China and will be useful in designing long-term, sustainable management strategies for SCMV.

## Introduction

Maize is one of the most important and widely cultivated food crops in the world [1–2]. USA is the leading producer of maize, followed closely by China. China produces about 30% of the world’s maize, amounting to 220 million tons in 2013. Within China, it is mainly grown in Jilin, Heilongjiang, Shanxi, Shandong, Hebei, Henan, Shaanxi, Sichuan, Hubei, and Hunan provinces [3–4]. In Shanxi alone, maize production was over 3 million tons in 2013 [5], valued at over $ 1.09 billion. Viral diseases pose a threat to maize production and cause economic losses [6]. Currently, three viruses have been reported to infect maize in Shanxi, among which *Sugarcane mosaic virus* (SCMV) is one of serious threat [7].

SCMV belongs to the genus *Potyvirus* within the family Potyviridae [8–9]. *Potyviruses* have a single-stranded positive-sense RNA genome. The genome of SCMV is approximately 9.6 kb long, covalently linked to a virus genome-linked protein at its 5′ terminus and poly (A) at its 3′ terminus [10]. The genome encodes a single large polyprotein, which is subsequently cleaved into 10 mature proteins (P1, HC-Pro, P3, 6K1, CI, 6K2, NIa-VPg, NIa-Pro, NIb, CP) by 3 self-encoded proteinases [10–11]. SCMV is easy to mutate because of the weak proofreading activity of RNA-dependent RNA polymerase, short generation time, and large population size [12–14]. As a consequence, the virus exists as numerous strains and replicates as complex and dynamic mutant swarms [14–15]. Understanding the genetic structure and the molecular variability factors of SCMV is not only an important aspect of evolutionary biology but also could be useful for virus management.

In recent years, numerous studies have been performed on the biology and genome characterization of SCMV worldwide [14–16]. One hundred and seventy-three SCMV isolates were grouped into five groups (sugarcane, maize, Thailand groups, the noble sugarcane and Brazil groups) based on CP gene sequences [17]. In further study, most of the codons of the CP gene proved to be under negative selection, and recombination also existed within the CP cistron [12]. The previous studies were based mainly on the CP gene due to the lack of whole genome sequences. In this study, SCMV isolates were collected from 4 regions (Xinzhou, Jinzhong, Linfen, and Yuncheng) in Shanxi during 2012 and 2013, and were tested by double-antibody sandwich (DAS)-ELISA and RT-PCR. The genomes of these SCMV were sequenced and compared with those available from online databases.

## Results

### Sequence Properties of SCMV Isolates

Double-antibody sandwich (DAS)-ELISA and RT-PCR showed 59 samples (30 samples in 2012 and 29 samples in 2013) to be positive for SCMV, of which 10 new isolates of SCMV were isolated and sequenced (Table 1). The incidences of SCMV collected from 4 regions (Xinzhou, Jinzhong, Linfen, and Yuncheng) in Shanxi, China were high up to 73.17% (30/41) and 64.44% (29/45) in 2012, 2013.

**Table 1:** ELISA and RT-PCR results for the samples collected from Shanxi, China.

Regions and geographic coordinates	2012			2013		
	Samples	ELISA	RT-PCR	Samples	ELISA	RT-PCR
Yuncheng: 111°31′–112°10′ E and 35°00′-35°39′ N, 110°59′–111°37′ E and 35°09′–35°34′ N, 111°02′–111°41′ E and 34°55′-35°19′ N	9	3^a^	6^b^	11	3^a^	7^b^
Jinzhong: 112°34′–113°8′ E and 37°23′–37°54′ N, 112°28′–113°01′ E and 37°12′–37°32′ N, 112°12′–112°31′ E and 37°12′–37°21′ N	11	4	8	13	6	9
Linfen: 111°05′–111°49′ E and 35°54′–36°19′ N, 111°06′–111°40′ E and 35°40′–36°03′ N, 111°30′–112°50′ E and 36°05′–36°23′ N	12	5	8	10	3	6
Xinzhou: 112°17′–112°58′ E and 38°13′–38°41′ N, 112°17′–113°35′ E and 38°35′– 39°09′ N, 112°39′–113°16′ E and 38°19′–38°40′ N	9	3	8	11	4	7
Total	41	15	30	45	16	29

^a^ The number of samples positive detected by ELISA for SCMV

^b^ **T**he number of samples positive detected by RT-PCR for SCMV

**Fig 1:**
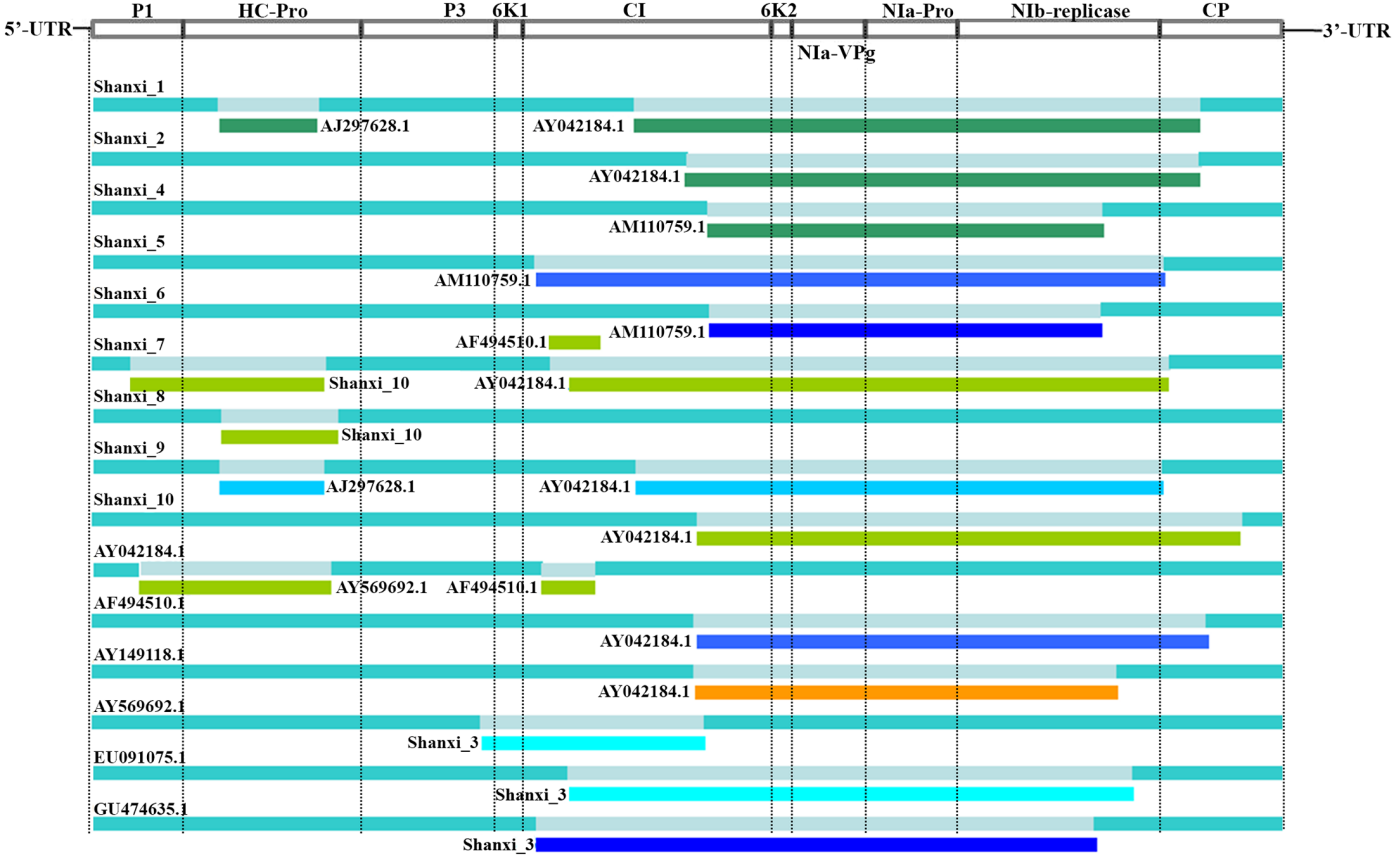
*Sugarcane mosaic virus* genome structure and potential recombination events. The RNA genome of SCMV, including the untranslated region (UTR) at the 5′ and 3′ termini (5′-UTR and 3′-UTR), was sequenced. The whole genome of SCMV (on the basis of 10 isolates) is 9610 nt long, including the 3′ and 5′ termini, and contains a single large open reading frame (ORF). Recombination events in the concatenated sequences of each SCMV isolate were identified by RDP3. Only the events supported by at least 5 different RDP3-implemented methods were considered and shown here. The 15 long light blue bars stand for the complete genomes of 15 recombinants while the 20 gray areas in the light blue bars represent regions substituted from another virus strain, identity thereof given by the 20 short differently colored bars corresponding to the gray areas.

The whole genome of SCMV, including the 3′ and 5′ termini, is 9610 nt long. It contained a single large open reading frame (ORF) (Fig 1). The putative ORF starts at AUG (148–150 nt). It encodes a polyprotein of 3,063 amino acids with an estimated molecular weight of 346.13 kDa. The polyprotein is subsequently processed into ten proteins (P1, HC-Pro, P3, 6K1, CI, 6K2, NIa-VPg, NIa-Pro, NIb, and CP) (Fig 1).

The polyprotein nucleotide and amino acid identity of 24 isolates (10 isolates from this study and 14 isolates from Genbank database) ranged from 79.06% to 100% and 88.95% to 100%, respectively. The highest identity was found between isolates KR611105 and KR611113 (nucleotide identity of 100%), while the identity between AY149118.1 and JX237862.1 was the lowest (nucleotide identity of 79.06%). At the amino acid level, the highest identity was found between isolates KR611105 and KR611113 (100%), while the identity between GU474635.1 and AJ278405.1 was the lowest (96.54%) (Table 2).

**Table 2:** Nucleotide and amino acid identity of *Sugarcane mosaic virus* isolates.

Gene	Nucleotide identity^a^	Amino acid identity
Polyprotein	79.06–100%	-
*P1*	67.05–100%	63.36–100%
*HC-Pro*	78.04–100%	92.61–100%
*P3*	77.96–100%	88.43–100%
*6K1*	74.13–100%	86.57–100%
*CI*	78.32–100%	94.36–100%
*6K2*	71.70–100%	71.70–100%
*NIa-Pro*	77.96–100%	88.43–100%
*NIb*	77.93–100%	90.98–100%
*NIa-VPg*	76.01–100%	86.77–100%
*CP*	82.03–100%	83.22–100%

^a^ The nucleotide and amino acid identity of SCMV isolates were calculated by Species Demarcation Tool (SDT) 1.0 software.

With regard to the genes, at the nucleotide level, the highest identity was found in the CP gene, with the identity ranging from 82.03% to 100%, followed by CI, and HC-Pro; P1 had the lowest identity (67.05–100%) (Table 2). At the amino acid level, the highest identity was found in CI, with the identity ranging from 94.36% to 100%, followed by HC-Pro, NIb, P3, and NIa-Pro, the lowest identity was found in P1 (63.36–100%) (Table 2).

### Nucleotide Sequence Similarities and Phylogenetic Analyses

**Fig 2:**
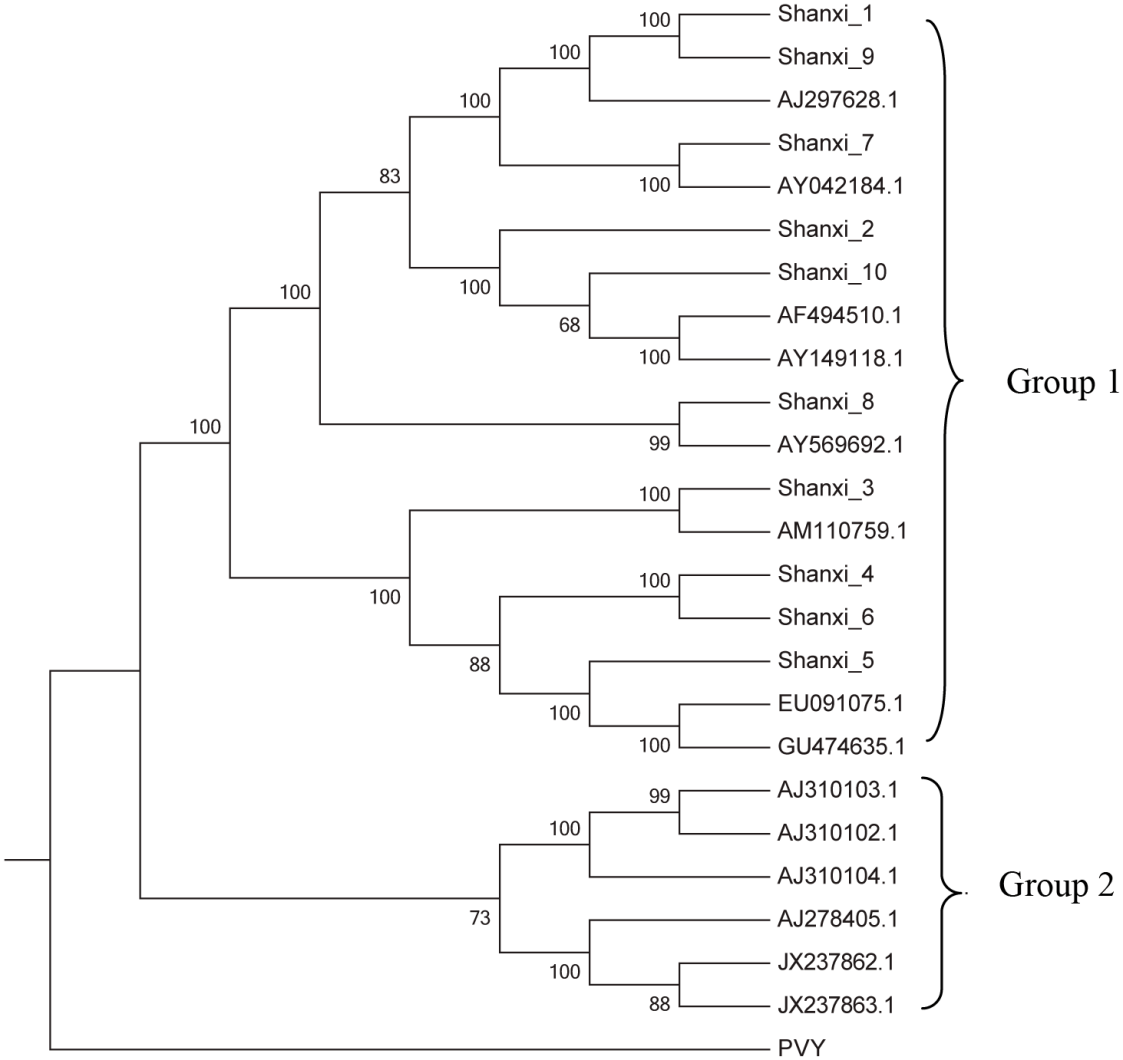
Neighbor-joining tree based on the genome sequences of *Sugarcane mosaic virus*. Bootstrap analysis was applied using 1000 replicates. Only bootstrap values (%) higher than 70 are given. *Potato virus Y* was used as the outgroup.

To further understand the genetic relationships among the global SCMV isolates, 24 isolates (10 isolates from this study and 14 isolates from Genbank database) were used for phylogenetic analysis. According to the phylogenetic tree, the 24 isolates were clustered into two groups (Fig 2). Group I included 18 isolates, all of which were isolated from maize collected from different sites (16 isolates from China, 2 isolates from Mexico). Group II contained 6 isolates, which were isolated from sugarcane from different regions spanning three continents (3 isolates from China, 2 isolates from Argentina, and 1 isolate from Australia). Nineteen isolates from China were classed into two different groups, while the isolates from maize grouped together. These results confirmed that the molecular diversity of SCMV isolates was closely associated with host species and not with geography.

Genetic distances within and between groups were calculated to determine the molecular diversity of the 24 SCMV isolates. The within-group genetic distances of group I and II were 0.0579 ± 0.0037 and 0.1907 ± 0.0115, respectively, and the inter-group genetic distance between group I and II was 0.1504 ± 0.0091 (Table 3). The inter-group genetic diversity was higher than the within-group genetic distances. This result suggested that it was host type rather than geography that played an important role in the genetic diversity of SCMV isolates.

**Table 3:** Estimates of evolutionary divergence and demographic trends within and between populations of *Sugarcane mosaic virus*.

Isolates	Genetic distance^a^	Tajima’s D	*F*_*ST*_^b^	*Snn*^c^
Group 1 and Group 1	0.0579 ± 0.0037	0.13187	-0.05882 (ns)^d^	0.00000 (ns)
Group 2 and Group 2	0.1907 ± 0.0115	0.99428	-0.20000 (ns)	0.00000 (ns)
Group 1 and Group 2	0.1504 ± 0.0091	0.06159	0.47328 (ns)	1.00000 (ns)

^a^ Evolutionary divergence and demographic trends were evaluated based on the pairwise analysis of CP gene sequences. Standard error values were obtained by a bootstrap procedure (1,000 replicates). Analyses were conducted in MEGA 5 using p-distance, and codon positions included were first, second, third, and non-coding. All positions containing gaps and missing data were eliminated from the data sets (complete deletion option). Tajima’s D statistics were calculated using DnaSP 5.10.01.

^b^ The *Fst* values were measured using DnaSP 5.10.01 to test the degree of differentiation among populations. The range 0.0–0.05 is considered to indicate little genetic differentiation; 0.05–0.15, moderate genetic differentiation; 0.15–0.25, heavy genetic differentiation; >0.25, complete genetic differentiation.

^c^ The nearest-neighbor statistic.

^d^ Probability (P value) obtained by permutation tests with 1,000 replicates, ns = not significant. All analyses were performed using DnaSP 5.10.01.

The *Fst* values (the interpopulational component of genetic variation, or the standardized variance in allele frequencies across populations) were measured to test the degree of differentiation among populations. Since the *Fst* values within groups were less than 0, the isolates within groups were highly similar and less differentiated among populations (Table 3). The *Fst* value between group 1 and 2 was as high as 0.25, which confirmed that the isolates between groups had a very high genetic differentiation (Table 3).

### Recombination Analysis and Selection Analysis

The potential recombination events in the genome sequences of 24 SCMV isolates were detected using the recombination detection program (RDP3) [18–20], and a total of 15 recombinant genomes resulting from 20 recombination events (Fig 2, Table 4).

**Table 4:** Potential recombination events of *Sugarcane mosaic virus* populations.

Recombinant	Supporting software	Major parent	Minor parent	p-value	Recombination site
Shanxi_1	**G**BMSC3^a^	Shanxi_10	AJ297628.1^b^	3.552 × 10^-8^	1040–1905
	GB**M**SC3	Shanxi_3	AY042184.1	3.896 × 10^-27^	4523–9216
Shanxi_2	GB**M**SC3	Shanxi_3	AY042184.1	3.896 × 10^-27^	4998–9216
Shanxi_4	GB**M**SC3	Shanxi_8	AM110759.1	2.861 × 10^-9^	5126–8406
Shanxi_5	GB**M**SC3	Shanxi_8	AM110759.1	2.861 × 10^-9^	3702–8865
Shanxi_6	GB**M**SC3	Shanxi_8	AM110759.1	2.861 × 10^-9^	5126–8406
Shanxi_7	GB**M**SC3	AJ297628.1	Shanxi_10	1.555 × 10^-9^	326–1938
	GB**M**SC3	Shanxi_3	AY042184.1	3.896 × 10^-27^	3941–8872
	GB**M**SC3	AJ297628.1	AF494510.1	4.523 × 10^-10^	3819–4260
Shanxi_8	**G**BMSC3	Shanxi_10	AJ297628.1	3.552 × 10^-8^	1040–2009
Shanxi_9	**G**BMSC3	Shanxi_10	AJ297628.1	3.552 × 10^-8^	1040–1950
	GB**M**SC3	Shanxi_3	AY042184.1	3.896 × 10^-27^	4523–8872
Shanxi_10	GB**M**SC3	Shanxi_3	AY042184.1	3.896 × 10^-27^	5025–9526
AY042184.1	GB**M**SC3	AJ297628.1	AY569692.1	2.601 × 10^-24^	364–1950
	GB**M**SC3	AJ297628.1	AF494510.1	4.523 × 10^-10^	3740–4188
AF494510.1	GB**M**SC3	Shanxi_3	AY042184.1	3.896 × 10^-27^	5000–9594
AY149118.1	GB**M**SC3	Shanxi_3	AY042184.1	3.896 × 10^-27^	5025–9594
AY569692.1	GBMS**C**3	AJ297628.1	Shanxi_3	6.850 × 10^-25^	3268–5124
EU091075.1	GB**M**SC3	Shanxi_8	Shanxi_3	3.481 × 10^-23^	3944–8593
GU474635.1	GB**M**SC3	Shanxi_8	Shanxi_3	3.481 × 10^-23^	3702–8406

^a^ Only events supported by at least 4 of the different RDP3-implemented methods were accepted. G: GENECONV; B: Bootscan; M: MaxChi; S: SiScan; C: Chimaera; 3: 3Seq. The highest significant p-value is marked in bold font and shown in the table.

^b^ GenBank accession number.

To further analyze selection pressure on the 24 SCMV isolates, the ratio between mutations in the non-synonymous and synonymous sites (d_N_/d_S_ ratio) were calculated. The d_N_ values for all genes of the 24 isolates were less than the d_S_ values (d_N_/d_S_ ratio < 1), which indicated that all the SCMV isolates were under negative selection (Table 5). In the polyprotein gene, 1085 sites were under negative selection (35.25%), with the maximum (282 sites) in CI and the minimum (7 sites) in 6K2 (Table 5).

**Table 5:** Estimates of selection pressure on genes of *Sugarcane mosaic virus* isolates.

Gene	Normalized dN-dS^a^		Positive sites^b^		Negative sites		Neutral sites	
	Log (L)	Mean (d_N_/d_S_)	Number	Percent	Number	Percent	Number	Percent
Polyprotein	-48070	0.1005	0	0	1085	35.25%	0	0
*P1*	-3575.3	0.1902	0	0	44	18.97%	0	0
*HC-Pro*	-5571.34	0.0319	0	0	172	37.39%	0	0
*P3*	-4262.13	0.0819	0	0	82	23.62%	0	0
*6K1*	-938.45	0.0756	0	0	28	41.79%	0	0
*CI*	-8497.46	0.0215	0	0	282	44.20%	0	0
*6K2*	-594.199	0.0816	0	0	7	13.21%	0	0
*NIa-Pro*	-2892.71	0.0456	0	0	76	31.40%	0	0
*NIb*	-6225.94	0.0483	0	0	171	31.82%	0	0
*VPG*	-2426.73	0.0686	0	0	60	31.75%	0	0
*CP*	-6587.8	0.2692	0	0	39	11.89%	0	0

^a^ Normalized values of the ratio of nonsynonymous substitutions per nonsynonymous site (d_N_) to synonymous substitutions per synonymous site (d_S_) (d_N_/d_S_) divided by the total length of the appropriate tree, a measure of selection pressure, was calculated for 24 SCMV isolates. Mean (d_N_/d_S_) value of <1 indicates negative or purifying selection; mean (d_N_/d_S_) = 1, neutral selection; and mean (d_N_/d_S_) > 1, positive selection, for each gene-specific sequence data set.

^b^ Positively or negatively selected sites are identified by at least 1 of the 3 selection pressure detection methods: single-likelihood ancestor counting (SLAC), fixed-effects likelihood (FEL), and internal fixed-effects likelihood (IFEL). SLAC is a counting method; FEL and IFEL are likelihood methods.

### Neutrality Tests and Population Demography

The mismatch distributions of SCMV were evaluated using concatenated sequences. The shapes of mismatch distributions of SCMV for all groups were multimodal and ragged (S1 and S2 Figs), indicating that all these populations were stable. Tajima’s *D* values for all SCMV populations were positive (Table 3), which supported that these populations were contracting. The p-values were not significant in any population. This result showed that the deduction might be less convincing (Table 4).

## Discussion

SCMV is a major threat to the maize production in China [12]. We collected SCMV populations and sequenced positive isolates of Shanxi in 2012 and 2013. These isolates showed different genotypes and formed different clades in the phylogenetic tree. Since the mismatch distributions of all SCMV isolates were multimodal and ragged (S1 and S2 Figs), all SCMV isolates had a trend of population diffusion. An increasing number of isolates with genetic differentiation might be generated in the field. Because of negative selection and recombination, genetic differentiation might be increasingly pronounced, and high-virulence strains might be generated in the field. This explains why SCMV exists in maize in the form of populations with ever-increasing molecular variability in the field. These potential high-virulence isolates are a potential threat to maize cultivated varieties, even those carrying resistance genes, because of the high possibility of overcoming the resistance genes. Considering the evolution mechanisms, an integrated management measures are needed to control SCMV, which should include breeding resistant cultivars, and controlling insect vectors (such as aphids) to prevent SCMV transmission to other crops.

According to the results, we can understand that SCMV isolated from maize is greatly different from SCMV isolated from Sugarcane. In China, maize is mainly grown in the north, while sugarcane is in the south. The differences of environment between north and south may generate the selection pressure on SCMV. Meanwhile, host is also one of the most important selection pressure. The genetic diversity of SCMV is mainly adapting to the complex and different conditions. Negative selection on the SCMV was also detected in this study. Under the negative selection, SCMV constantly accumulated the available variations to adapt to the difference of conditions.

For RNA viruses, recombination is a natural phenomenon and played an important role in evolution. Recombination events have been reported in SCMV. Based on the CP gene of SCMV, six recombinants were detected [12]. Although the field study was not carried out to test the potential recombination events, it at least showed that recombination was a natural phenomenon for SCMV. Recombination may play an important role in the evolution of SCMV, and be an important reason for SCMV genetic diversity.

The conservation of ten genes of SCMV differed from each other. As a member of the genus *Potyvirus*, the complete RNA genome of SCMV encodes a large polyprotein, which is processed into 10 mature proteins by 3 virus-encoded proteases after translation [10–11]. These 10 proteins play different roles in the life cycle of SCMV (infection, replication, movement, and transmission) [21–24]. We found that the nucleotide identity in the CP gene was the highest, followed by CI, HC-Pro, and P3, the lowest nucleotide identity was found in P1. This may be attributed to the various roles of these genes during the SCMV life cycle. The phylogenetic trees of 24 SCMV isolates were also diverse when based on different genes, which confirmed that it was more accurate to study genetic diversity of SCMV based on the whole genome than on one gene or partial sequence. In addition, the probability of recombination was diverse for different genes. Most of recombination events were found in 6K2, NIa-VPg, NIa-Pro and CI gene, while some were in P1 and CP genes (Fig 1). These results showed that the genes which are near 5′ and 3′ termini were more conserved, and recombination may be an important reason for the phenomenon.

Taken together, our results demonstrated that SCMV isolates formed two divergent evolutionary groups. Host, negative selection and recombination were found to be the important evolutionary factors shaping the genetic structure of these SCMV populations. Using infectious clones of SCMV should facilitate the study of gene function and biological characteristics. Our findings provide a foundation for evaluating the epidemiological characteristics of SCMV in China and will be useful in designing long-term, sustainable management strategies for SCMV.

## Materials and Methods

### Sample Collection in the Field

In 2012 and 2013, the incidence survey of SCMV were conducted in 4 different regions (Xinzhou, Jinzhong, Linfen, and Yuncheng) in Shanxi, China. 86 samples were collected from different cultivation areas: 41 samples in 2012 and 45 samples in 2013 (permitted by the Shanxi Plant Protection and Plant Quarantine Station). The location and number of the samples are listed in Table 1.

### Design and Selection of Primers

Five complete sequences of SCMV isolates (GenBank ID: AJ297618.1, AM110759.1, AY042184.1, AY149118.1, and EU091075.1) were obtained from NCBI and analyzed by DNAMAN 6.0. Based on the conserved domains, primers were designed to detect SCMV by Primer Premier 5.0. The primer sequences, size and position of amplification products expected are listed in Table 6.

**Table 6:** Primer sequences, the size and position of amplification products expected from the *Sugarcane mosaic virus* genome.

Primer	Sequence (5′–3′)	Product (bp)	Position
SCMV-F1	TAGTGAACGGCTCGGTAGGA	1787	70–1856
SCMV-R1	CTTGGTGGTGTTGTGTTTGG	1787	70–1856
SCMV-F2	CATGTTGCTGCGTTACAACGG	1509	1724–3232
SCMV-R2	TTGCTTCAATGAGGCGTGGGT	1509	1724–3232
SCMV-F3	ATCTGACATGCGATCAGTTT	1856	3170–5025
SCMV-R3	TGTAGGGCTGTATTGAAGGA	1856	3170–5025
SCMV-F4	GACTGAAGGTCATAACGCAC	1374	4655–6048
SCMV-R4	TACCCTCATACTCTGGGAAG	1374	4655–6048
SCMV-F5	AGAGCACTTCAGCGTCATCAGA	1711	5829–7539
SCMV-R5	CAAACGGTTCCACCCACCATAA	1711	5829–7539
SCMV-F6	GACTCGGACTTTTACAGCAG	1966	7392–9357
SCMV-R6	AACAGGGTTTCCAGGAGACT	1966	7392–9357
3′ RACE-Inner	GGCGAGACTCAGGAGAATAC	-	9250–9269
3′ RACE-Outer	ACCACTAGTCTCCTGGAAAC	-	9332–9351
5′ RACE-Inner	GCAAGCCTTTGTTCCTCAGTGT	-	254–275
5′ RACE-Outer	CACTAGTGACACAAGCCTTCC	-	296–316

### RT-PCR, Cloning, and Sequencing

RNA was extracted from samples using the Universal Plant Total RNA Extraction Kit (DP405-02, BioTeke, China), and cDNA was synthesized using the Prime Script RT reagent Kit (D6130, TaKaRa, Japan). PCR was carried out in a 25-μL PCR mixture including 2 μL of cDNA template, 2.5 μL of 25 mM Mg^2+^ (M2101, Promega, USA), 2.5 μL of a dNTP mixture with each dNTP at 5 mM, 2.5 μL of 10× polymerase buffer (M2101, Promega, USA), 0.5 μL of 5 U/μL Hot-start Taq polymerase (M2101, Promega, USA), and 2 μL sense and antisense primers (10 μM each). The reaction process was as follows: denaturation at 94°C for 3 min; 35 cycles of denaturation at 94°C for 30 s, primer annealing at 52°C for 1 min, and primer extension at 72°C for 2 min; and final extension at 72°C for 10 min. For the 5′-terminal and 3′-terminal sequence, 5′ RACE and 3′ RACE reactions were conducted using the 5′ RACE and 3′ RACE system (D315, TaKaRa, Japan). The size of PCR products was examined by 2% agarose gel under UV light. The positive bands were purified from the agarose gel using a gel extraction kit (DP204-02, BioTeke, China). These fragments were inserted into a pGEM-T simple vector and cloned into *Escherichia coli* JM109. For each fragment, at least 3 clones from each ligation were sequenced. If there was any difference at any position of the sequences, at least 4 clones were sequenced to obtain the consensus sequence.

Specific PCR primers were designed for primer walking and obtaining the fragment sequences. The complete nucleotide sequences of all SCMV isolates were generated based on the fragment sequences and SCMV genome sequences deposited in the GenBank database using ClustalX program [25]. The GenBank accession numbers of all SCMV isolate genome sequences are listed in S1 Table.

### Recombination and Phylogenetic Analysis

The high-similarity sequences of the SCMV isolates were selected for further analysis by BLAST (http://www.ncbi.nlm.nih.gov/BLAST/). Multiple alignments of nucleotide sequences and corresponding amino acid sequences were performed using MultAlin (http://bioinfo.genotoul.fr/multalin/multalin.html) [26]. The recombination analysis of these SCMV isolates based on the whole genome was carried out using the recombination detection program (RDP3) [18]. The 7 methods (RDP, GENECONV, BOOTSCAN, MAXCHI, CHIMAERA, SISCAN, and 3SEQ) implemented in RDP were used in the recombination analysis [18–20]. An event detected by at least 5 different methods and with p-values < 10^−6^ was considered to be a positive recombination event [18–20, 27].

Phylogenetic relationships were determined by neighbor-joining in MEGA 5 [27]. Bootstrap analysis with 1,000 replicates was performed to evaluate the significance of the internal branches. Branches with less than 70% bootstrap value were collapsed.

### Analysis of Genetic Distance and Selection Pressure

The genetic distances of SCMV isolates within and between groups were calculated by the maximum composite likelihood method in MEGA 5 [27]. The selection pressure was estimated by the d_N_/d_S_ ratio, where d_N_ represented the average number of non-synonymous substitutions per non-synonymous site and d_S_ represented the average number of synonymous substitutions per synonymous site. The values of d_N_ and d_S_ were estimated using the PBL method in MEGA 5 [27]. The gene is under positive (or diversifying) selection when the d_N_/d_S_ ratio is >1, neutral selection when d_N_/d_S_ ratio = 1, and negative (or purifying) selection when d_N_/d_S_ ratio < 1 [28].

### Demography Analyses

Tajima’s *D* statistical test was performed to analyze the population changes in SCMV by DnaSP 5.0 [29–30]. Tajima’s D measures the departure from neutrality for all mutations in a genomic region. The purpose of the test is to distinguish randomly and non-random process for a DNA sequence. In the mismatch distribution, a smooth unimodal Poisson distribution indicated that the population had a star-like phylogeny due to the accumulation of low-frequency mutations during a recent expansion; ragged multimodal distributions indicated that the population was experiencing long-term demographic stability [28].

## Supporting Information

S1 Fig Mismatch distributions of 18 *Sugarcane mosaic virus* isolates in group 1 were calculated using DnaSP 5. (TIF)

S2 Fig Mismatch distributions of 6 *Sugarcane mosaic virus* isolates in group 2 were calculated using DnaSP 5. (TIF)

S1 Table Information of 10 *Sugarcane mosaic virus* isolates analyzed (GenBank accession numbers KR611105–KR611114). (DOC)
