# Supplementary figures and images for: Molecular Variability and Distribution of Sugarcane Mosaic Virus in Shanxi, China
Source: PLoS One. 2016 Mar 17;11(3):e0151549. doi: 10.1371/journal.pone.0151549 (PMC4795778; doi:10.1371/journal.pone.0151549)

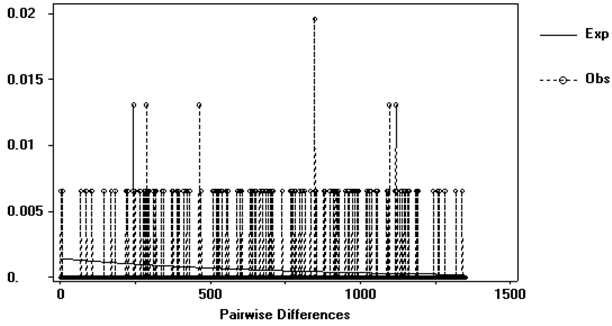

Supplement: S1 Fig — (TIF) [file pone.0151549.s001.tif]

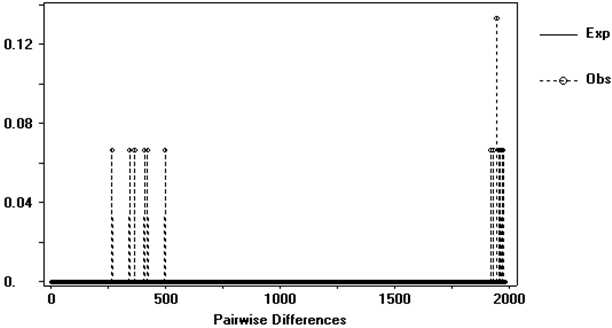

Supplement: S2 Fig — (TIF) [file pone.0151549.s002.tif]
