# Supplementary material for: Molecular Variability and Distribution of Sugarcane Mosaic Virus in Shanxi, China
Source: PLoS One. 2016 Mar 17;11(3):e0151549. doi: 10.1371/journal.pone.0151549 (PMC4795778; doi:10.1371/journal.pone.0151549)
Supplement: S1 Table — (DOC) [file pone.0151549.s003.doc]

**Table S1.** The information of GenBank accession number KR611105-KR611114

| Isolates | Date of collection | Geographical origin | Accession number |
| --- | --- | --- | --- |
| Shanxi­_1 | 02-AUG-2012 | Yuncheng, China | KR611105 |
| Shanxi­_2 | 03-AUG-2013 | Yuncheng, China | KR611106 |
| Shanxi­_3 | 05-Aug-2012 | Linfen, China | KR611107 |
| Shanxi­_4 | 06-Aug-2012 | Linfen, China | KR611108 |
| Shanxi­_5 | 06-Aug-2012 | Linfen, China | KR611109 |
| Shanxi­_6 | 09-Aug-2013 | Jinzhong, China | KR611110 |
| Shanxi­_7 | 10-Aug-2012 | Jinzhong, China | KR611111 |
| Shanxi­_8 | 10-Aug-2013 | Jinzhong, China | KR611112 |
| Shanxi­_9 | 12-Aug-2012 | Xinzhou, China | KR611113 |
| Shanxi­_10 | 13-Aug-2013 | Xinzhou, China | KR611114 |
